# Genotypic investigation of a rotavirus cluster at a quaternary-care pediatric hospital

**DOI:** 10.1017/ice.2022.317

**Published:** 2023-10

**Authors:** Eimear M. Kitt, Hee-won Yoon, Courtney E. Comar, Kenneth P. Smith, Rebecca M. Harris, Mathew D. Esona, Rashi Gautam, Slavica Mijatovic-Rustempasic, Amy L. Hopkins, Jose Jaimes, Lori K. Handy

**Affiliations:** 1 Department of Infection Prevention and Control, Children’s Hospital of Philadelphia, Philadelphia, Pennsylvania; 2 Division of Infectious Diseases, Children’s Hospital of Philadelphia, Philadelphia, Pennsylvania; 3 Perelman School of Medicine, University of Pennsylvania, Philadelphia, Pennsylvania; 4 Clinical Microbiology Laboratory, Hospital of the University of Pennsylvania, Philadelphia, Pennsylvania; 5 Infectious Diseases Diagnostics Laboratory, Children’s Hospital of Philadelphia, Philadelphia, Pennsylvania; 6 Viral Gastroenteritis Branch, Division of Viral Diseases, National Center for Immunization and Respiratory Diseases (NCIRD), Centers for Disease Control and Prevention (CDC), Atlanta, Georgia

## Abstract

Rotavirus (RV) was a common healthcare-associated infection prior to the introduction of the RV vaccine. Following widespread RV vaccination, healthcare-associated rotavirus cases are rare. We describe an investigation of a cluster of rotavirus infections in a pediatric hospital in which an uncommon genotype not typically circulating in the United States was detected.

Group A rotavirus (RVA) was among the most common healthcare-associated infections (HAIs) in industrialized countries prior to introduction of the rotavirus vaccines.^1^ RotaTeq (RV5; Merck, Rahway, NJ) was introduced in 2006, providing protection for any G (VP7) 1, 2, 3, 4 or P (VP4) 1[8] serotypes. Rotarix (RV1; GlaxoSmithKline, Philadelphia, PA) was introduced in 2008 and provided protection for any G (VP7) 1 or P (VP4) 1[8] serotype. In addition to a substantial reduction in hospital admissions and medical costs related to acute gastroenteritis (AGE),^2^ there has been a large decrease in nosocomial acquisition of RVA in the post-vaccine period in regions with widespread vaccination.^3^

In the spring of 2022, through routine surveillance, we identified a cluster of rotavirus cases in a population of pediatric patients in 2 adjacent inpatient units. Given the increased incidence from baseline at our institution, which is typically 1–5 infections per year, and detection in an older pediatric population of whom at least 50% were fully vaccinated against RVA, we collaborated with the Centers for Disease Control and Prevention (CDC) to perform an outbreak investigation that included RVA genotyping. All patients had epidemiologic links by contiguous bed spaces or shared care teams. Sequencing was conclusive for 9 of the 10 stool samples and was determined to be a G9P[4] genotype, which is rarely detected among children in the United States. Here, we describe the collaborative epidemiologic response that took place, and we highlight some key learnings from our investigation.

## Methods

The Children’s Hospital of Philadelphia (CHOP) is a 594-bed, free-standing, quaternary-care, pediatric hospital that serves as the local community hospital as well as a regional, national, and global referral center. CHOP has 28,000 admissions per year, and ∼40% of inpatient beds are in intensive care units. This work met CHOP predetermined standards under which work does not require review of the institutional review board and is considered exempt.

The Department of Infection Prevention and Control at CHOP performs routine house-wide surveillance for all HAIs using CDC National Healthcare Safety Network surveillance definitions. Potential healthcare-associated viral gastrointestinal (GI) infections are identified from positive clinical specimens using routine gastrointestinal virus molecular testing data, complemented by a review of the electronic health record. Healthcare-associated cases are defined as onset of infection-specific symptoms on or after day 3 of admission or within 1 calendar day of discharge. Specific to rotavirus, cases were considered unrelated to vaccine administration when the virus was detected by PCR 1 month or longer after vaccination. According to the CHOP protocol, cluster infection control procedures were implemented on the unit when 3 or more cases among employees, patients or both, were identified by microbiologic testing, in this case gastrointestinal PCR testing, or symptom reporting in a 48-hour period. Due to the rarity of rotavirus clusters, and the increased incidence from baseline that was observed, we established a partnership with CDC to obtain sequencing and instituted additional local infection control measures.

RVA-positive stool samples from patients with acute gastroenteritis were submitted to the CDC for RVA strain genotyping and characterization. RVA strains were genotyped using the genotype-specific qRT-PCR assays for VP7 and VP4 genes. Next-generation sequencing (NGS) was performed for RVA strain characterization by sequencing the cDNA libraries on Illumina MiSeq.^4^ NGS data were analyzed using the reference-based and denovo assembly on CLC Genomics Workbench 21 software (http://www.clcbio.com/products/clc-genomics-workbench/). Genotypes were determined according to guidelines of the Rotavirus Classification Working Group^5^ and using the NCBI BLASTN program. Nomenclature is such that no parentheses or brackets are used for the VP7 gene (G genotype), however, the VP4 gene denotes the P genotype expressed as ‘P’ with the bracket denoting a strain identified by RT-PCR genotyping and confirmed via gene sequencing, thus written as G9P[4].

## Results

**Fig. 1.**
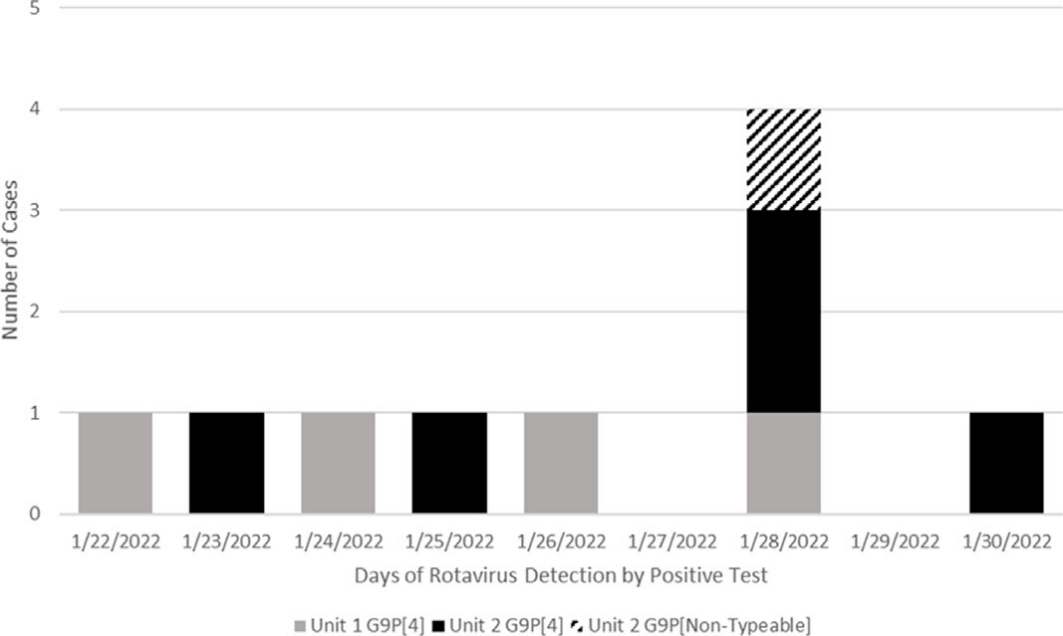
Rotavirus infections in the affected unit at the Children’s Hospital in Philadelphia, by day of positive test.

Epidemiologic surveillance identified an RVA cluster involving 10 patients aged between 10 months to 10 years on 2 adjacent inpatient units. The sample cluster was 60% male, with a median age of 1.8 years (interquartile range, 0.7–10). A significant proportion of these patients were medically complex, with 80% documented to have chronic respiratory failure requiring ventilation at baseline. Of the infected patients, 50% were documented to have been vaccinated for rotavirus with either Rotateq or Rotarix. Symptoms included emesis, diarrhea, or both. All patients had epidemiologic links by contiguous bed spaces or shared care teams. Sequencing was conclusive for 9 of the 10 stool samples to be a G9P[4] genotype (Fig. 1). Local infection control measures implemented as our hospital’s response to clusters included just-in-time education for employees, increased cleaning using Oxivir-TB, use of either single-patient rooms or placing RVA-positive patients in cohorts with ongoing use of contact precautions, use of soap and water on room exit, and furlough of symptomatic healthcare workers. Ongoing transmission ceased following these measures. All patients recovered with supportive care and no escalation of care was needed. Review of the cases later revealed a parent with GI symptoms as the possible index case, though this was not confirmed by testing. Notably, 15 employees between the affected units reported gastrointestinal symptoms; however diagnostic testing is not routinely performed on healthcare workers. Additionally, norovirus was circulating in the community, and not all employees with AGE were epidemiologically linked to the patient or to other employees. Ongoing work is being done to sequence banked rotavirus-positive specimens collected prior to the outbreak from the community to investigate when the outbreak strain was introduced into the region.

## Discussion

### Increased incidence of RVA HAI

Our outbreak investigation revealed an increased incidence in HAI infections due to RVA compared to baseline, in an older pediatric population who were documented to be at least 50% fully vaccinated against rotavirus. Notably, G9P[4] genotype identified in outbreak patients is a nonvaccine rotavirus strain not typically endemic to the United States. In children, protection from RVA infection is mediated by neutralizing antibodies that target epitopes on VP4, VP7, or both.^6^ We suspect that the high incidence noted by surveillance in our institution was most likely multifactorial, including in part from lack of immune protection from the vaccine. Our current approved RVA vaccination platform does not include direct coverage for either of the VP4 (P[4]) or VP7 (G9) proteins found in this strain. Cross-serotypic protection for multiple homotypic or heterotypic rotavirus strains has been described with both Rotarix and RotaTeq vaccines, which has contributed to the global success of the rotavirus vaccines.^7^ Cross protection has been described for the G9 genotype family in general, however, admittedly not for G9P[4] specifically.^8^ In addition, we postulate that there was a large contribution from the so-called ‘immune debt’ that many populations experienced during the social isolation of the COVID-19 pandemic, in that the significant decrease in circulating pathogens contributed to reduced immune protection to common childhood pathogens, including common diseases like rotavirus. Validation of this hypothesis would require further investigation both in this population and in children at large.^9^

### Emergence of G9P[4] genotype

The G9P[4] genotype is admittedly rare among children in the United States. It is primarily detected in surveillance in Latin America and was first identified in Brazil in the 1990s.^10^ Recent surveillance data suggest that this particular genotype is part of an uncommon group of G- and P- combinations that together represent <2% of all RVA strains in the United States.^11^ The rarity of the strain and likelihood that much of the US population was not previously exposed likely contributed to more symptomatic individuals in our setting. It is unknown whether this genotype will continue to emerge, but the timing of its introduction to the local community will be important to fully investigate. Ongoing surveillance after the licensure of vaccine continues to be important in vaccinated populations.

### Outbreak measures

Our collaborative effort identified an unusual genotype of RVA in our quaternary-care pediatric hospital. Despite the aforementioned ‘immune debt’ and rarity of the strain, transmission halted with the persistent use of our healthcare-associated viral infection bundle, which included the following: local infection control measures implemented during any cluster response of increased education and cleaning, isolation of RVA-positive patients in single rooms or placing patients in cohorts in semiprivate rooms with ongoing use of contact precautions, use of soap and water on room exit, and furlough of symptomatic healthcare workers.

This study had several limitations. Lack of diagnostic testing on symptomatic AGE healthcare workers as well as concomitant gastrointestinal viruses including norovirus circulating in the local community and hospital setting, meant that we were unable to definitively link employees epidemiologically to our affected patients. Given the extent of symptomatic individuals, it is therefore possible that we underestimated the extent of the outbreak or may have had an unidentified healthcare worker as an index case. As described above, our additional work of sequencing banked rotavirus positive specimens should shed some light on when the outbreak strain was introduced into the region, and the current proportion attributed to this particular genotype.

In conclusion, routine surveillance of healthcare-associated GI illness led to identification of a cluster of infections; RVA strain genotyping and characterization identified unusual rotavirus genotype G9P[4] as the cause. Partnership between hospital epidemiology, the institution’s laboratory, and the CDC revealed the need to implement standard infection prevention cluster response measures on the affected units to halt ongoing transmission as well as pursue genotyping of community samples to better characterize current epidemiology of rotavirus in a highly vaccinated population.
